# 2-(1,3-Benzodioxol-5-yl)-1*H*-benzimid­azole

**DOI:** 10.1107/S1600536814000737

**Published:** 2014-01-18

**Authors:** Nadir Ghichi, Mohamed Amine Benaouida, Ali Benosmane, Ali Benboudiaf, Hocine Merazig

**Affiliations:** a Unité de Recherche de Chimie de l’Environnement et Moléculaire Structurale (CHEMS), Faculté des Sciences Exactes, Département de Chimie, Université Constantine 1, Algeria

## Abstract

The asymmetric unit of the title compound, C_14_H_10_N_2_O_2_, contains two independent mol­ecules. In each mol­ecule, the benzodioxole ring system displays an envelope conformation, with the methyl­ene C atom located at the flap deviating by 0.081 (2) and 0.230 (2) Å from the mean plane formed by the other atoms. The dihedral angles between the benzo­imidazole ring system (all atoms) and the benzodioxole benzene ring are 15.35 (6) and 10.99 (7)°. In the crystal, mol­ecules are linked by N—H⋯N hydrogen bonds into chains running along the [101].

## Related literature   

For the biological activity of imidazole derivatives and their use as inhibitors of neurodegenerative disorders and as anti­tumor drugs, see: Park *et al.* (1977 ▶). For related imidazole compounds, see: Andreani *et al.* (2005 ▶); Xu *et al.* (2010 ▶).
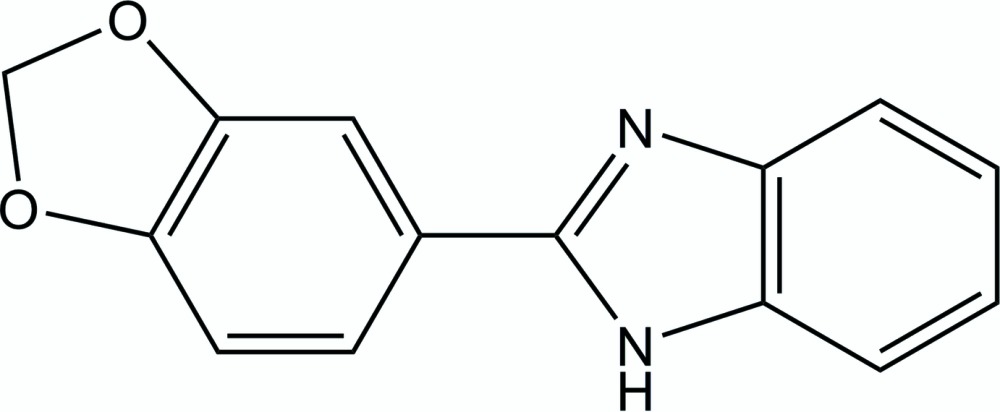


## Experimental   

### 

#### Crystal data  

C_14_H_10_N_2_O_2_
*M* *_r_* = 238.24
Monoclinic, 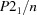

*a* = 8.7454 (7) Å
*b* = 15.2824 (11) Å
*c* = 16.9487 (13) Å
β = 91.974 (5)°
*V* = 2263.9 (3) Å^3^
*Z* = 8
Mo *K*α radiation
μ = 0.10 mm^−1^
*T* = 293 K
0.03 × 0.02 × 0.01 mm

#### Data collection  

Nonius KappaCCD diffractometer
13101 measured reflections
4003 independent reflections
3422 reflections with *I* > 2σ(*I*)
*R*_int_ = 0.018

#### Refinement  

*R*[*F* ^2^ > 2σ(*F* ^2^)] = 0.034
*wR*(*F* ^2^) = 0.088
*S* = 1.02
4003 reflections
325 parameters
H-atom parameters constrained
Δρ_max_ = 0.19 e Å^−3^
Δρ_min_ = −0.18 e Å^−3^

### 

Data collection: *KappaCCD Server Software* (Nonius, 1999 ▶); cell refinement: *KappaCCD Server Software*; data reduction: *DENZO* and *SCALEPACK* (Otwinowski & Minor, 1997 ▶); program(s) used to solve structure: *SHELXS97* (Sheldrick, 2008 ▶); program(s) used to refine structure: *SHELXL97* (Sheldrick, 2008 ▶); molecular graphics: *ORTEP-3 for Windows* (Farrugia, 2012 ▶); software used to prepare material for publication: *SHELXL97*.

## Supplementary Material

Crystal structure: contains datablock(s) global, I. DOI: 10.1107/S1600536814000737/xu5756sup1.cif

Structure factors: contains datablock(s) I. DOI: 10.1107/S1600536814000737/xu5756Isup2.hkl

Supporting information file. DOI: 10.1107/S1600536814000737/xu5756Isup3.cml

CCDC reference:

Additional supporting information:  crystallographic information; 3D view; checkCIF report

## Figures and Tables

**Table 1:** Hydrogen-bond geometry (Å, °)

*D*—H⋯*A*	*D*—H	H⋯*A*	*D*⋯*A*	*D*—H⋯*A*
N1—H1*N*⋯N4^i^	0.86	1.93	2.7761 (15)	168
N3—H3*N*⋯N2	0.86	1.96	2.8053 (16)	168

Symmetry code: (i) 
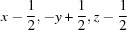
.

**Fig. 1.**
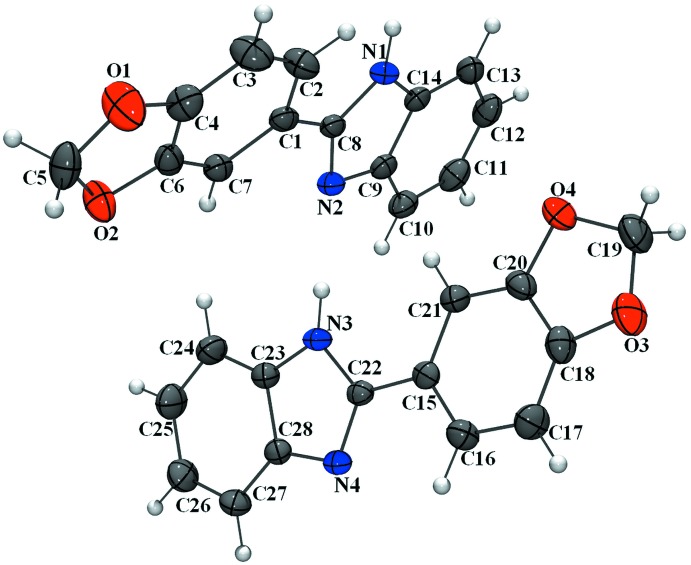
View of the molecular structure of the title compound, with atom labelling. Displacement ellipsoids are drawn at the 50% probability level.

**Fig. 2.**
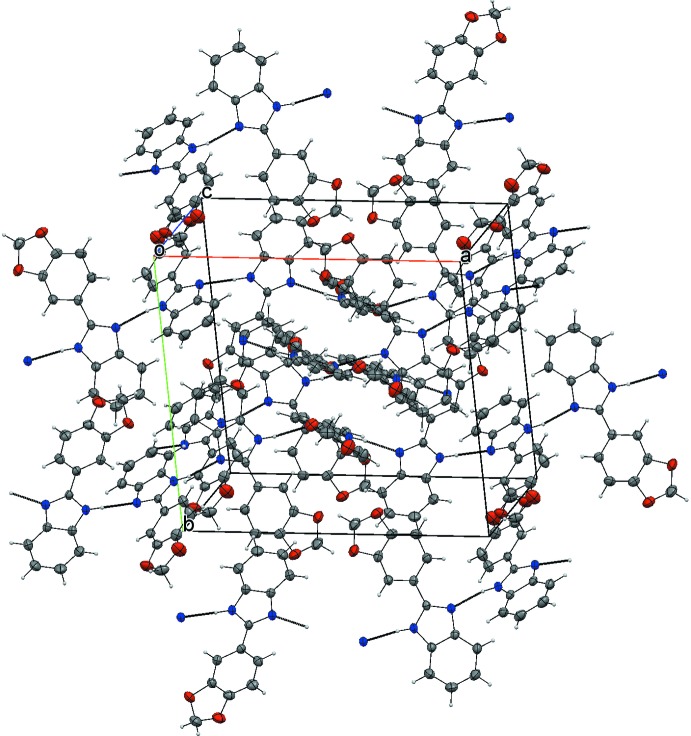
Partial view along the b axis of the crystal packing of the title compound, showing the hydrogen bonds as dashed lines (see Table 1 for details).

## supplementary crystallographic information

### 1. Comment

Owing to the promising biological activities as inhibitors of neurodegenerative disorders and antitumor drugs (Park *et al.*, 1977), most of these investigations were carried out with imidazole (Andreani *et al.*, 2005; Xu *et al.*, 2010).

A view of the molecular structure of (I) with numbering Scheme is Shown in Fig1. In the crystal, intermolecular N—H···N hydrogen bond Fig2, link the molecules into chains running along the [101] direction.

### 2. Experimental

A mixture of 1,2-diaminebenzene (2.3 g) and benzo[*d*][1,3]dioxole-5-carbaldehyde (0.02 mole) in ethanol (60 ml) was refluxed for 6 h. The yellow crystals obtained are filter and rinsed by a mixture of water ice ethanol, the recrystalization is done in excess of chloroform in 15 days.

### 3. Refinement

The H atoms attached to C atoms and N atom were fixed geometrically and treated as riding with C—H = 0.93 Å (aromatic) or 0.97 Å (methylene) and N—H = 0.86 Å with U_iso_(H) = 1.2U_eq_(C,N).

### Crystal data

**Table:**

C_14_H_10_N_2_O_2_	*F*(000) = 992
*M**_r_* = 238.24	*D*_x_ = 1.398 Mg m^−^^3^
Monoclinic, *P*2_1_/*n*	Mo *K*α radiation, λ = 0.71073 Å
Hall symbol: -P 2yn	Cell parameters from 5620 reflections
*a* = 8.7454 (7) Å	θ = 2.5–25.1°
*b* = 15.2824 (11) Å	µ = 0.10 mm^−^^1^
*c* = 16.9487 (13) Å	*T* = 293 K
β = 91.974 (5)°	Prism, yellow
*V* = 2263.9 (3) Å^3^	0.03 × 0.02 × 0.01 mm
*Z* = 8	

### Data collection

**Table:**

Nonius KappaCCD diffractometer	3422 reflections with *I* > 2σ(*I*)
Radiation source: fine-focus sealed tube	*R*_int_ = 0.018
Graphite monochromator	θ_max_ = 25.1°, θ_min_ = 1.8°
CCD rotation images, thick slices scans	*h* = −10→10
13101 measured reflections	*k* = −18→18
4003 independent reflections	*l* = −18→20

### Refinement

**Table:**

Refinement on *F*^2^	Primary atom site location: structure-invariant direct methods
Least-squares matrix: full	Secondary atom site location: difference Fourier map
*R*[*F*^2^ > 2σ(*F*^2^)] = 0.034	Hydrogen site location: inferred from neighbouring sites
*wR*(*F*^2^) = 0.088	H-atom parameters constrained
*S* = 1.02	*w* = 1/[σ^2^(*F*_o_^2^) + (0.0405*P*)^2^ + 0.8431*P*] where *P* = (*F*_o_^2^ + 2*F*_c_^2^)/3
4003 reflections	(Δ/σ)_max_ = 0.001
325 parameters	Δρ_max_ = 0.19 e Å^−^^3^
0 restraints	Δρ_min_ = −0.18 e Å^−^^3^

### Special details

**Table:**

Geometry. All esds (except the esd in the dihedral angle between two l.s. planes) are estimated using the full covariance matrix. The cell esds are taken into account individually in the estimation of esds in distances, angles and torsion angles; correlations between esds in cell parameters are only used when they are defined by crystal symmetry. An approximate (isotropic) treatment of cell esds is used for estimating esds involving l.s. planes.
Refinement. Refinement of F^2^ against ALL reflections. The weighted R-factor wR and goodness of fit S are based on F^2^, conventional R-factors R are based on F, with F set to zero for negative F^2^. The threshold expression of F^2^ > 2sigma(F^2^) is used only for calculating R-factors(gt) etc. and is not relevant to the choice of reflections for refinement. R-factors based on F^2^ are statistically about twice as large as those based on F, and R- factors based on ALL data will be even larger.

### Fractional atomic coordinates and isotropic or equivalent isotropic displacement parameters (Å^2^)

**Table:**

	*x*	*y*	*z*	*U*_iso_*/*U*_eq_	
N1	0.16720 (13)	0.28274 (7)	0.48294 (6)	0.0245 (3)	
H1N	0.1584	0.2651	0.4348	0.029*	
N2	0.27132 (13)	0.34182 (7)	0.59250 (6)	0.0248 (3)	
N3	0.46175 (13)	0.33066 (7)	0.72874 (7)	0.0268 (3)	
H3N	0.4134	0.3292	0.6837	0.032*	
N4	0.59892 (13)	0.28499 (7)	0.83388 (6)	0.0251 (3)	
O1	0.83709 (13)	0.38755 (9)	0.37149 (7)	0.0532 (3)	
O2	0.79659 (12)	0.45860 (7)	0.48949 (7)	0.0413 (3)	
O3	0.51495 (14)	−0.07450 (7)	0.63889 (7)	0.0452 (3)	
O4	0.36723 (14)	0.03150 (7)	0.57597 (6)	0.0439 (3)	
C1	0.43467 (16)	0.33554 (9)	0.47685 (8)	0.0248 (3)	
C2	0.46177 (17)	0.29304 (10)	0.40626 (9)	0.0330 (3)	
H2	0.3889	0.2543	0.3855	0.040*	
C3	0.59527 (18)	0.30704 (11)	0.36603 (9)	0.0405 (4)	
H3	0.6133	0.2787	0.3187	0.049*	
C4	0.69848 (17)	0.36405 (10)	0.39901 (9)	0.0342 (4)	
C5	0.89677 (19)	0.45149 (10)	0.42461 (11)	0.0414 (4)	
H5A	0.9041	0.5075	0.3980	0.050*	
H5B	0.9984	0.4346	0.4437	0.050*	
C6	0.67386 (16)	0.40628 (9)	0.46969 (9)	0.0290 (3)	
C7	0.54339 (15)	0.39333 (9)	0.51010 (8)	0.0260 (3)	
H7	0.5273	0.4217	0.5577	0.031*	
C8	0.29278 (15)	0.32083 (8)	0.51781 (7)	0.0219 (3)	
C9	0.12289 (16)	0.31528 (9)	0.60706 (8)	0.0250 (3)	
C10	0.03920 (17)	0.32189 (9)	0.67524 (8)	0.0316 (3)	
H10	0.0810	0.3474	0.7209	0.038*	
C11	−0.10750 (18)	0.28935 (10)	0.67265 (9)	0.0380 (4)	
H11	−0.1653	0.2928	0.7176	0.046*	
C12	−0.17177 (18)	0.25144 (10)	0.60455 (10)	0.0374 (4)	
H12	−0.2712	0.2299	0.6052	0.045*	
C13	−0.09151 (16)	0.24510 (9)	0.53635 (9)	0.0308 (3)	
H13	−0.1348	0.2202	0.4908	0.037*	
C14	0.05686 (15)	0.27748 (8)	0.53860 (8)	0.0242 (3)	
C15	0.53562 (15)	0.17347 (9)	0.73203 (8)	0.0239 (3)	
C16	0.63003 (16)	0.11017 (9)	0.76669 (9)	0.0301 (3)	
H16	0.6944	0.1257	0.8092	0.036*	
C17	0.63104 (18)	0.02407 (10)	0.73960 (10)	0.0365 (4)	
H17	0.6943	−0.0181	0.7631	0.044*	
C18	0.53483 (17)	0.00446 (9)	0.67688 (9)	0.0329 (3)	
C19	0.3884 (2)	−0.06069 (11)	0.58423 (10)	0.0455 (4)	
H19A	0.4097	−0.0866	0.5335	0.055*	
H19B	0.2967	−0.0875	0.6039	0.055*	
C20	0.44481 (17)	0.06728 (10)	0.64038 (8)	0.0304 (3)	
C21	0.44051 (17)	0.15187 (9)	0.66618 (8)	0.0293 (3)	
H21	0.3776	0.1933	0.6414	0.035*	
C22	0.53328 (15)	0.26200 (9)	0.76528 (7)	0.0227 (3)	
C23	0.48053 (16)	0.40285 (9)	0.77669 (8)	0.0272 (3)	
C24	0.43107 (19)	0.48911 (10)	0.76926 (9)	0.0371 (4)	
H24	0.3734	0.5080	0.7254	0.045*	
C25	0.4717 (2)	0.54506 (10)	0.83015 (10)	0.0415 (4)	
H25	0.4415	0.6033	0.8271	0.050*	
C26	0.5574 (2)	0.51643 (10)	0.89636 (9)	0.0395 (4)	
H26	0.5823	0.5561	0.9364	0.047*	
C27	0.60577 (18)	0.43110 (10)	0.90375 (9)	0.0339 (3)	
H27	0.6622	0.4124	0.9481	0.041*	
C28	0.56718 (16)	0.37349 (9)	0.84232 (8)	0.0256 (3)	

### Atomic displacement parameters (Å^2^)

**Table:**

	*U*^11^	*U*^22^	*U*^33^	*U*^12^	*U*^13^	*U*^23^
N1	0.0275 (6)	0.0278 (6)	0.0181 (5)	−0.0007 (5)	−0.0025 (5)	−0.0013 (4)
N2	0.0284 (6)	0.0265 (6)	0.0194 (6)	0.0001 (5)	−0.0019 (5)	0.0003 (4)
N3	0.0318 (7)	0.0279 (6)	0.0203 (6)	0.0001 (5)	−0.0069 (5)	0.0008 (5)
N4	0.0279 (6)	0.0270 (6)	0.0203 (6)	0.0001 (5)	−0.0022 (5)	0.0006 (5)
O1	0.0384 (7)	0.0707 (9)	0.0516 (7)	−0.0127 (6)	0.0180 (6)	−0.0126 (6)
O2	0.0320 (6)	0.0425 (6)	0.0496 (7)	−0.0109 (5)	0.0057 (5)	−0.0056 (5)
O3	0.0553 (7)	0.0301 (6)	0.0501 (7)	−0.0003 (5)	0.0000 (6)	−0.0116 (5)
O4	0.0605 (8)	0.0354 (6)	0.0348 (6)	−0.0073 (5)	−0.0099 (5)	−0.0088 (5)
C1	0.0265 (7)	0.0248 (7)	0.0228 (7)	0.0036 (5)	−0.0038 (6)	0.0024 (5)
C2	0.0298 (8)	0.0402 (9)	0.0288 (8)	−0.0007 (6)	−0.0008 (6)	−0.0069 (6)
C3	0.0381 (9)	0.0535 (10)	0.0303 (8)	−0.0005 (8)	0.0063 (7)	−0.0128 (7)
C4	0.0285 (8)	0.0408 (9)	0.0335 (8)	0.0036 (6)	0.0077 (7)	0.0021 (7)
C5	0.0340 (8)	0.0338 (8)	0.0573 (11)	−0.0026 (7)	0.0140 (8)	0.0051 (7)
C6	0.0269 (7)	0.0246 (7)	0.0353 (8)	−0.0002 (6)	−0.0024 (6)	0.0032 (6)
C7	0.0282 (7)	0.0255 (7)	0.0242 (7)	0.0020 (6)	0.0001 (6)	0.0003 (5)
C8	0.0260 (7)	0.0199 (6)	0.0194 (7)	0.0018 (5)	−0.0033 (5)	0.0019 (5)
C9	0.0281 (7)	0.0229 (7)	0.0240 (7)	0.0031 (5)	−0.0007 (6)	0.0049 (5)
C10	0.0403 (9)	0.0314 (8)	0.0233 (7)	0.0018 (6)	0.0029 (6)	0.0024 (6)
C11	0.0394 (9)	0.0394 (9)	0.0359 (9)	0.0033 (7)	0.0115 (7)	0.0090 (7)
C12	0.0283 (8)	0.0397 (9)	0.0446 (9)	−0.0017 (7)	0.0041 (7)	0.0129 (7)
C13	0.0288 (8)	0.0306 (8)	0.0326 (8)	−0.0028 (6)	−0.0041 (6)	0.0058 (6)
C14	0.0270 (7)	0.0220 (7)	0.0235 (7)	0.0020 (5)	−0.0012 (6)	0.0050 (5)
C15	0.0227 (7)	0.0277 (7)	0.0216 (7)	−0.0025 (5)	0.0041 (5)	0.0006 (5)
C16	0.0279 (7)	0.0321 (8)	0.0300 (8)	0.0015 (6)	−0.0016 (6)	−0.0029 (6)
C17	0.0366 (8)	0.0306 (8)	0.0421 (9)	0.0084 (6)	−0.0011 (7)	−0.0016 (7)
C18	0.0375 (8)	0.0276 (8)	0.0342 (8)	−0.0019 (6)	0.0079 (7)	−0.0050 (6)
C19	0.0595 (11)	0.0351 (9)	0.0415 (10)	−0.0096 (8)	−0.0008 (8)	−0.0115 (7)
C20	0.0344 (8)	0.0337 (8)	0.0232 (7)	−0.0068 (6)	0.0011 (6)	−0.0035 (6)
C21	0.0349 (8)	0.0283 (7)	0.0246 (7)	−0.0012 (6)	−0.0022 (6)	0.0020 (6)
C22	0.0222 (7)	0.0271 (7)	0.0188 (7)	−0.0010 (5)	0.0018 (5)	0.0019 (5)
C23	0.0302 (8)	0.0277 (7)	0.0236 (7)	−0.0012 (6)	−0.0009 (6)	0.0009 (6)
C24	0.0443 (9)	0.0301 (8)	0.0362 (9)	0.0030 (7)	−0.0072 (7)	0.0037 (6)
C25	0.0521 (10)	0.0268 (8)	0.0456 (10)	0.0031 (7)	−0.0006 (8)	−0.0020 (7)
C26	0.0526 (10)	0.0322 (8)	0.0337 (9)	−0.0036 (7)	0.0001 (8)	−0.0085 (6)
C27	0.0411 (9)	0.0364 (8)	0.0239 (7)	−0.0030 (7)	−0.0043 (6)	−0.0027 (6)
C28	0.0282 (7)	0.0267 (7)	0.0218 (7)	−0.0021 (6)	0.0009 (6)	0.0005 (5)

### Geometric parameters (Å, º)

**Table:**

N1—C8	1.3599 (17)	C9—C14	1.4025 (19)
N1—C14	1.3749 (17)	C10—C11	1.375 (2)
N1—H1N	0.8600	C10—H10	0.9300
N2—C8	1.3256 (17)	C11—C12	1.393 (2)
N2—C9	1.3902 (18)	C11—H11	0.9300
N3—C22	1.3599 (17)	C12—C13	1.376 (2)
N3—C23	1.3768 (18)	C12—H12	0.9300
N3—H3N	0.8600	C13—C14	1.388 (2)
N4—C22	1.3261 (17)	C13—H13	0.9300
N4—C28	1.3890 (18)	C15—C16	1.389 (2)
O1—C4	1.3619 (18)	C15—C21	1.4080 (19)
O1—C5	1.416 (2)	C15—C22	1.4660 (19)
O2—C6	1.3709 (17)	C16—C17	1.394 (2)
O2—C5	1.4335 (19)	C16—H16	0.9300
O3—C18	1.3760 (18)	C17—C18	1.366 (2)
O3—C19	1.434 (2)	C17—H17	0.9300
O4—C20	1.3784 (17)	C18—C20	1.375 (2)
O4—C19	1.427 (2)	C19—H19A	0.9700
C1—C2	1.389 (2)	C19—H19B	0.9700
C1—C7	1.4017 (19)	C20—C21	1.366 (2)
C1—C8	1.4600 (19)	C21—H21	0.9300
C2—C3	1.389 (2)	C23—C24	1.392 (2)
C2—H2	0.9300	C23—C28	1.3981 (19)
C3—C4	1.361 (2)	C24—C25	1.377 (2)
C3—H3	0.9300	C24—H24	0.9300
C4—C6	1.384 (2)	C25—C26	1.398 (2)
C5—H5A	0.9700	C25—H25	0.9300
C5—H5B	0.9700	C26—C27	1.375 (2)
C6—C7	1.3652 (19)	C26—H26	0.9300
C7—H7	0.9300	C27—C28	1.396 (2)
C9—C10	1.3926 (19)	C27—H27	0.9300
			
C8—N1—C14	107.59 (11)	C12—C13—C14	117.08 (14)
C8—N1—H1N	126.2	C12—C13—H13	121.5
C14—N1—H1N	126.2	C14—C13—H13	121.5
C8—N2—C9	105.23 (11)	N1—C14—C13	132.81 (13)
C22—N3—C23	107.80 (11)	N1—C14—C9	105.36 (12)
C22—N3—H3N	126.1	C13—C14—C9	121.83 (13)
C23—N3—H3N	126.1	C16—C15—C21	119.89 (13)
C22—N4—C28	105.45 (11)	C16—C15—C22	119.74 (12)
C4—O1—C5	106.09 (12)	C21—C15—C22	120.35 (12)
C6—O2—C5	105.20 (12)	C15—C16—C17	121.91 (14)
C18—O3—C19	104.80 (12)	C15—C16—H16	119.0
C20—O4—C19	104.78 (12)	C17—C16—H16	119.0
C2—C1—C7	120.50 (13)	C18—C17—C16	116.92 (14)
C2—C1—C8	120.70 (13)	C18—C17—H17	121.5
C7—C1—C8	118.80 (12)	C16—C17—H17	121.5
C3—C2—C1	121.48 (14)	C17—C18—C20	121.65 (14)
C3—C2—H2	119.3	C17—C18—O3	128.39 (14)
C1—C2—H2	119.3	C20—C18—O3	109.91 (13)
C4—C3—C2	117.00 (14)	O4—C19—O3	107.66 (12)
C4—C3—H3	121.5	O4—C19—H19A	110.2
C2—C3—H3	121.5	O3—C19—H19A	110.2
C3—C4—O1	127.87 (14)	O4—C19—H19B	110.2
C3—C4—C6	122.23 (14)	O3—C19—H19B	110.2
O1—C4—C6	109.90 (14)	H19A—C19—H19B	108.5
O1—C5—O2	108.56 (12)	C21—C20—C18	122.58 (14)
O1—C5—H5A	110.0	C21—C20—O4	127.65 (14)
O2—C5—H5A	110.0	C18—C20—O4	109.76 (13)
O1—C5—H5B	110.0	C20—C21—C15	116.98 (13)
O2—C5—H5B	110.0	C20—C21—H21	121.5
H5A—C5—H5B	108.4	C15—C21—H21	121.5
C7—C6—O2	128.50 (13)	N4—C22—N3	111.92 (12)
C7—C6—C4	121.62 (13)	N4—C22—C15	124.77 (12)
O2—C6—C4	109.87 (13)	N3—C22—C15	123.32 (12)
C6—C7—C1	117.17 (13)	N3—C23—C24	132.47 (13)
C6—C7—H7	121.4	N3—C23—C28	105.24 (12)
C1—C7—H7	121.4	C24—C23—C28	122.30 (13)
N2—C8—N1	112.32 (12)	C25—C24—C23	116.66 (14)
N2—C8—C1	124.57 (12)	C25—C24—H24	121.7
N1—C8—C1	123.11 (11)	C23—C24—H24	121.7
N2—C9—C10	130.30 (13)	C24—C25—C26	121.64 (15)
N2—C9—C14	109.49 (12)	C24—C25—H25	119.2
C10—C9—C14	120.21 (13)	C26—C25—H25	119.2
C11—C10—C9	117.62 (14)	C27—C26—C25	121.64 (14)
C11—C10—H10	121.2	C27—C26—H26	119.2
C9—C10—H10	121.2	C25—C26—H26	119.2
C10—C11—C12	121.76 (14)	C26—C27—C28	117.67 (14)
C10—C11—H11	119.1	C26—C27—H27	121.2
C12—C11—H11	119.1	C28—C27—H27	121.2
C13—C12—C11	121.50 (14)	N4—C28—C27	130.30 (13)
C13—C12—H12	119.3	N4—C28—C23	109.59 (12)
C11—C12—H12	119.3	C27—C28—C23	120.09 (13)

### Hydrogen-bond geometry (Å, º)

**Table:**

*D*—H···*A*	*D*—H	H···*A*	*D*···*A*	*D*—H···*A*
N1—H1*N*···N4^i^	0.86	1.93	2.7761 (15)	168
N3—H3*N*···N2	0.86	1.96	2.8053 (16)	168

Symmetry code: (i) *x*−1/2, −*y*+1/2, *z*−1/2.
